# Supplementary material for: Cadherins orchestrate specific patterns of perisomatic inhibition onto distinct pyramidal cell populations
Source: Nat Commun. 2025 May 14;16:4481. doi: 10.1038/s41467-025-59635-z (PMC12078473; doi:10.1038/s41467-025-59635-z)
Supplement: Supplementary file 1 — Supplementary Information [file 41467_2025_59635_MOESM1_ESM.pdf]

Cadherins orchestrate specific patterns of perisomatic inhibition onto distinct pyramidal cell populations

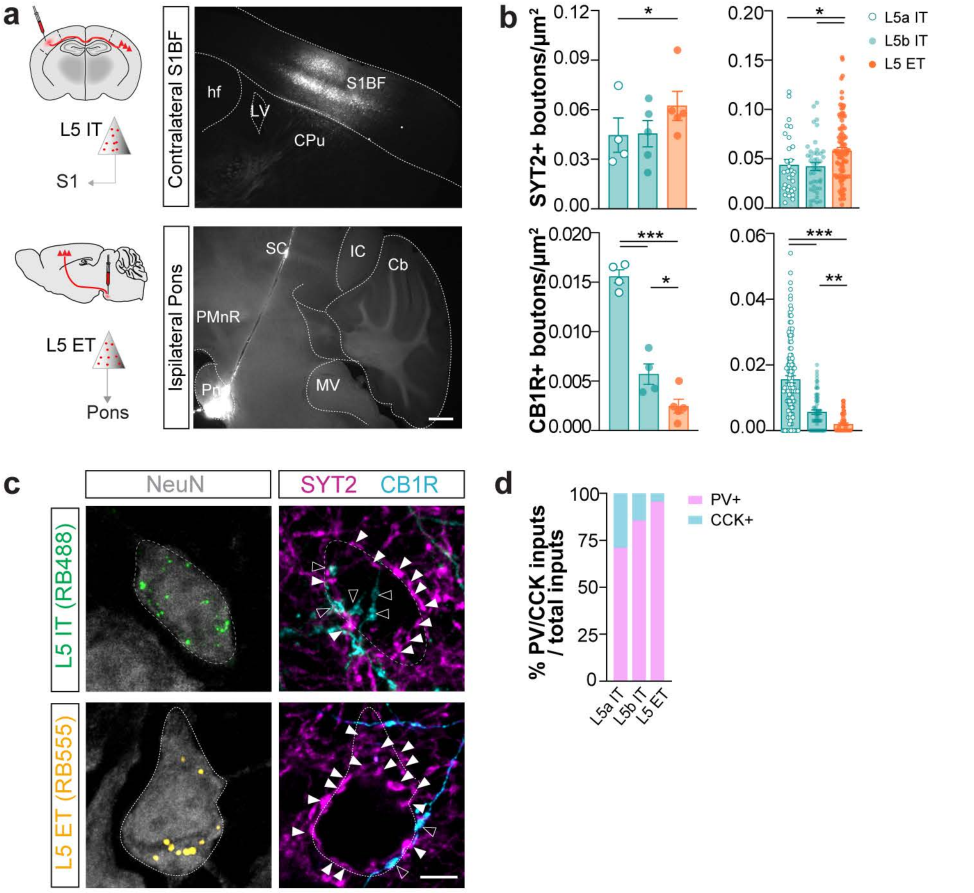

Supplementary Figure 1: L5 pyramidal cell populations exhibit different ratios of PV+ and CCK+ inhibition

a Confocal images of retrobeads injection site in the barrel field area of the somatosensory cortex and the pons to target L5 IT and L5 ET neurons. **b** SYT2+ boutons density at P30 across L5 sub-layers (L5a IT *n* = 4 mice, 30 cells; L5b IT *n* = 5 mice, 44 cells; L5 ET *n* = 5 mice, 97 cells; mouse analysis: Mixed-effects model F(0.8919, 3.122) = 8.078, Tukey's posthoc: L5a IT vs L5b IT *p* = 0.9969, L5a IT vs L5 ET *p = 0.0461, L5b IT vs L5 ET *p* = 0.0581; cell analysis: Kruskal-Wallis test **p = 0.0054, Dunn's posthoc: L5a IT vs L5b IT *p*> 0.9999, L5a IT vs L5 ET *p = 0.0479, L5b IT vs L5 ET *p = 0.0191). CB1R+ boutons density at P30 across L5 sub-layers (L5a IT *n* = 4 mice, 120 cells; L5b IT *n* = 4 mice, 65 cells; L5 ET *n* = 3 mice, 52 cells; mouse analysis: One-way ANOVA F= 71.72, ***p< 0.000, Tukey's posthoc: L5a IT vs L5b IT ***p< 0.0001, L5a IT vs L5 ET ***p< 0.0001, L5b IT vs L5 ET *p = 0.0386; cell analysis: Kruskal-Wallis test ***p< 0.0001, Dunn's posthoc: L5a IT vs L5b IT ***p< 0.0001, L5a IT vs L5 ET ***p< 0.0001, L5b IT vs L5 ET *p = 0.0081). **c** Maximum projections of PV (white arrows) and CCK (open white arrows) inputs onto L5 IT and L5 ET somas. **d** Fraction of PV+ and CCK+ inputs over total perisomatic inputs in L5 IT and L5 ET neurons at P30 (L5a IT PV= 70,96% CCK = 29.04%, 77 cells, 2 mice; L5b IT PV= 85,73% CCK = 14.27%, 32 cells, 2 mice; L5 ET PV = 95,67% CCK = 4.33%, 44 cells, 3 mice). Cerebellum (Cb); Caudate putamen (CPu); Hippocampal formation (hf); Inferior colliculus (IC); Paramedian raphe nucleus (PMrR); Medial vestibular nuclei (MV); Pontine nucleus (Pn); Primary somatosensory cortex, Barrel Field (S1BF); Superior colliculus (SC); Lateral ventricle (LV). Scale bars: 500µm (**a**), 5µm (**b**). Data are represented as mean ± SEM. Source data are provided as a Source Data file.

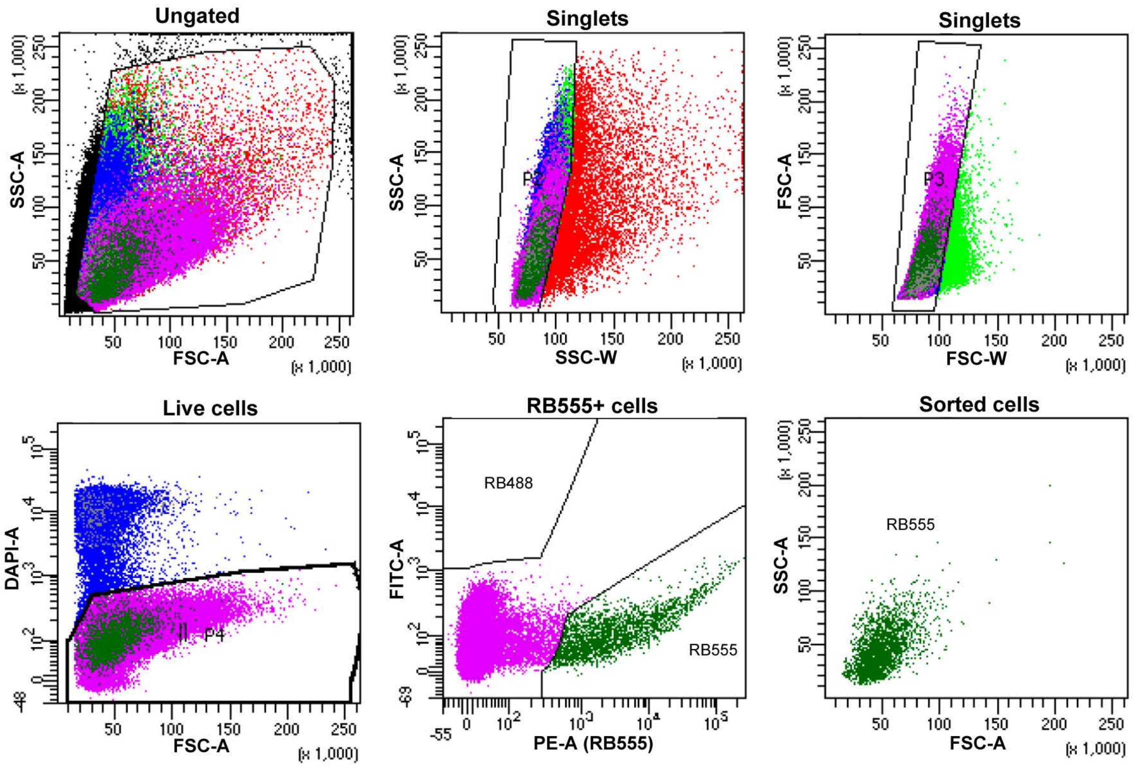

Supplementary Figure 2: Fluorescence-Activated Cell Sorting gating strategy

Parameters and sequential gating steps used to sort L5 IT and L5 ET neurons retrogradely labelled with red fluorescent retrobeads (RB555+). The fraction of live RB555+ neurons was gated as PE-A+/DAPI-. SSC: Side scatter area; FSC-A: Forward scatter area; SSC-W: Side scatter width; FSC-W: Forward scatter width; DAPI-A: 4',6-diamidino-2-phenylindole area; FITC-A: Fluorescein isothiocyanate area; PE-A: phycoerythrin area.

a

samples	reads	% uniq mapped	detected genes	% mRNA	% junctions
L5 IT 1	140 182 987	84.9	22 108	73.7	47.0
L5 IT 2	129 784 379	87.3	22 450	73.5	50.4
L5 IT 3	145 713 901	87.4	22 190	73.7	51.3
L5 ET 1	133 762 013	86.0	21 626	74.8	51.5
L5 ET 2	131 227 323	89.5	22 528	73.5	50.5
L5 ET 3	131 233 918	88.6	22 011	73.8	52.5

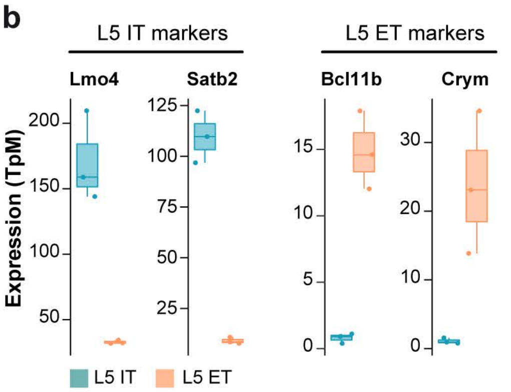

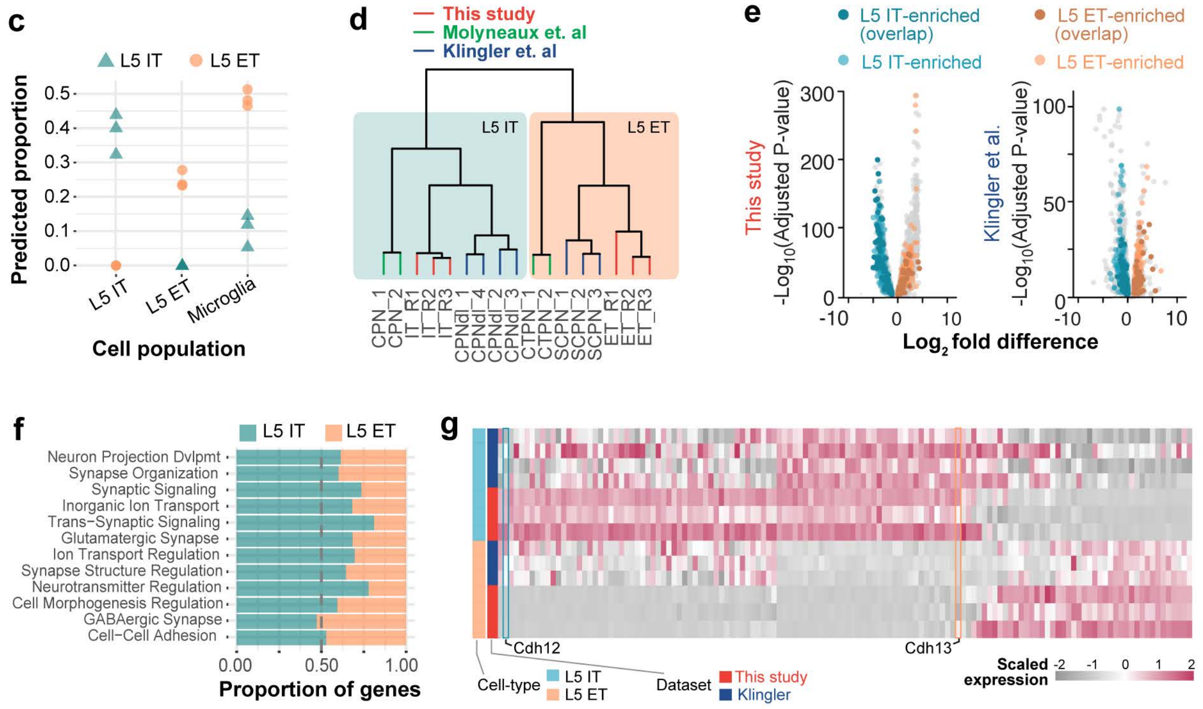

Supplementary Figure 3: Complementary information about the RNA-sequencing data analysis
a FastQC analysis of RNA sequencing data for each experimental replicate showing the total number of reads and the percentage of reads uniquely mapped to the reference genome, number of detected genes, mRNA representation and reads mapped to exon-exon junctions. **b** Expression levels (TpM) of key markers genes in L5 IT and L5 ET pyramidal cell types. **c** Estimated cell type proportions (L5 IT, L5 ET and microglia) after deconvolution of our RNAseq dataset. **d** Dendrogram of this study, Molyneaux et al., and Klinger et al., with branches annotated by experimental replicate and L5 pyramidal cell types. Note the low distance among replicates and how samples cluster by L5 pyramidal cell type. **e** Volcano plot of DEGs in our study and Klingler et al. **f** Proportion of gene ontology terms enriched in L5 IT or L5 ET cell types. **g** Heatmap showing DEG between L5 IT and L5 ET neurons in this study and Klingler et al.

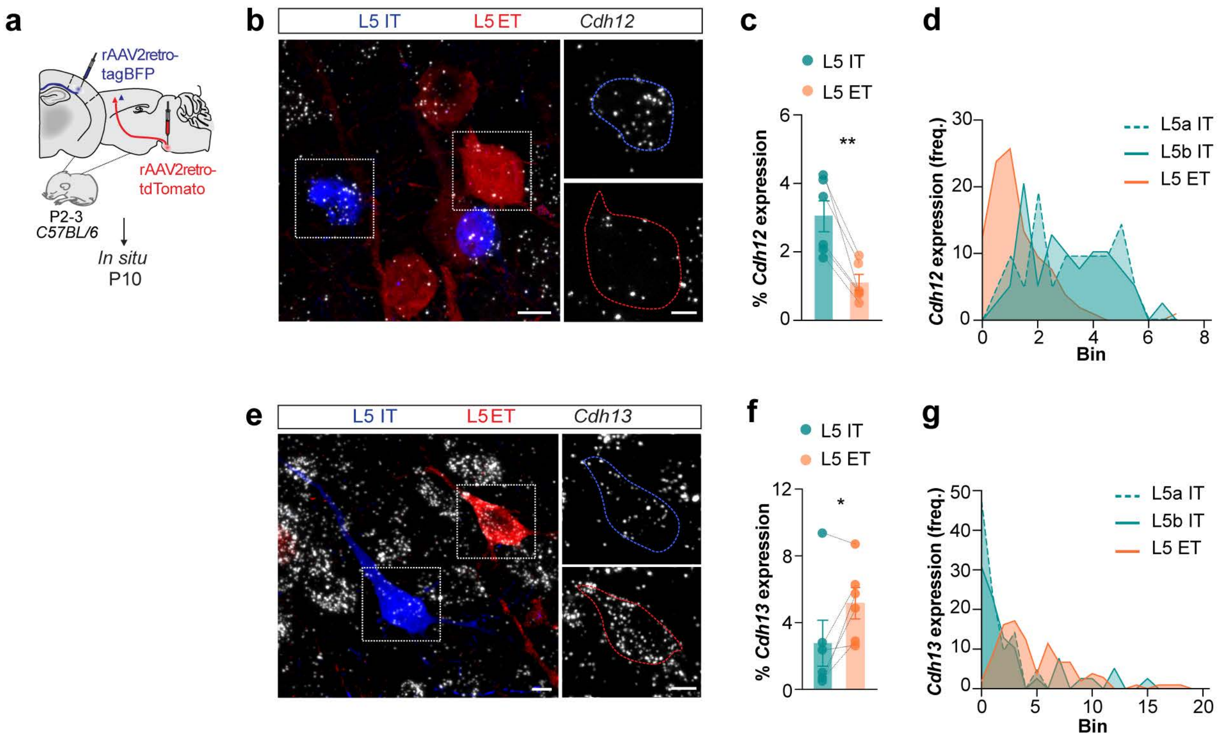

Supplementary Figure 4: *In situ* validation of candidate genes enrichment in L5 IT and L5 ET neurons
a Experimental strategy. **b, e** Representative images of *Cdh12* and *Cdh13* RNA expression in retrogradely-labeled L5 IT and L5 ET neurons. **c** *Cdh12* expression in L5 IT and L5 ET neurons (n = 6 mice; two-sided paired t-test **p = 0.0026). **d** Frequency distribution of *Cdh12* expression across L5a IT, L5b IT and L5 ET neurons. **f** *Cdh13* expression in L5 IT and L5 ET neurons (n = 6 mice; two-sided paired t-test *p = 0.0477). **g** Frequency distribution of *Cdh13* expression across L5a IT, L5b IT and L5 ET neurons. Each dot represents an individual mouse. Scale bars: 10µm, 5µm (**b, e**). Source data are provided as a Source Data file.

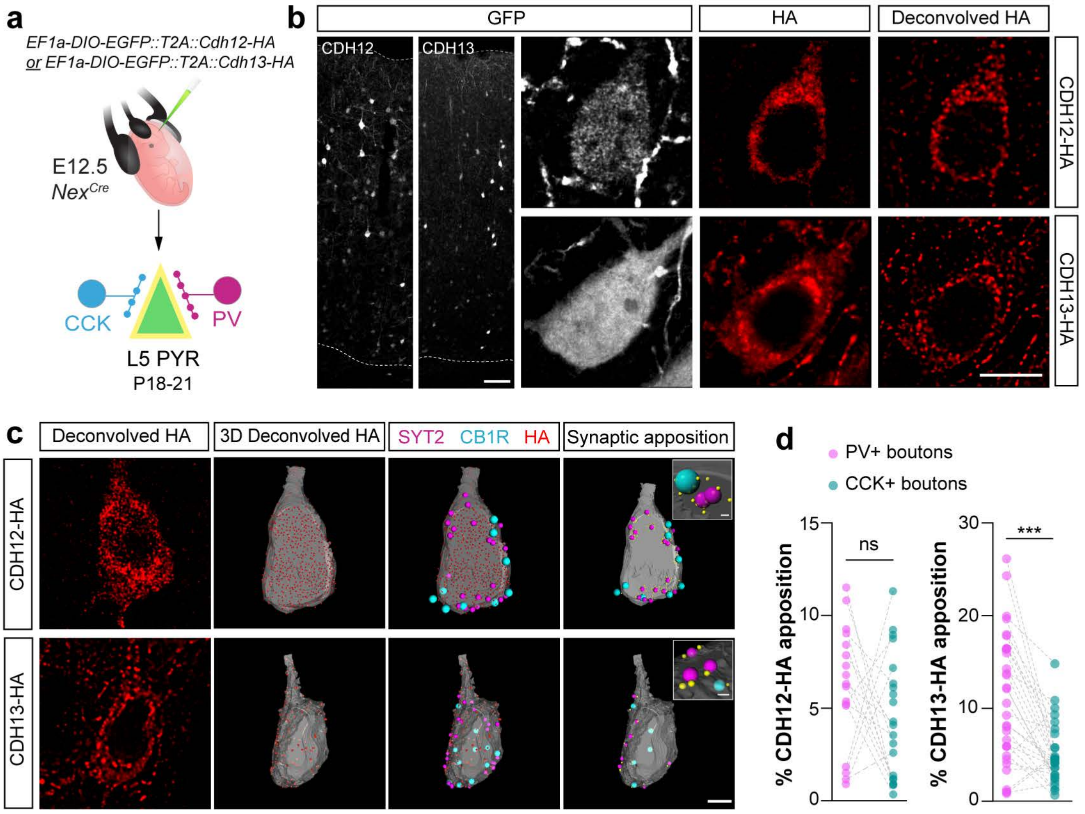

Supplementary Figure 5: CDH12 and CDH13 protein localization

a In utero electroporation strategy in *Nex*^{Cre} mice. Cre-dependent HA-tagged *Cdh12* and *Cdh13* constructs were overexpressed in *Nex*^{Cre} mice in order to assess cadherin protein localization in L5 pyramidal neurons at P18-21. **b** Representative images of electroporated pyramidal neurons (cytosolic GFP+) depicting membrane expression of HA-tagged CDH12 or CDH13 proteins. Signal deconvolution unraveled a dense clustered expression of HA-CDH12 and HA-CDH13 at the soma of GFP+ L5 pyramidal neurons. **c** Representative 3D-reconstructed images of CDH12-HA or CDH13-HA expression at the surface of L5 pyramidal cell somas. A fraction of post-synaptic CDH12-HA and CDH13-HA (red dots) are in close apposition (yellow dots, insert images) to PV (SYT2+, magenta dots) and CCK boutons (CB1R+, cyan dots). **d** Fraction of post-synaptic CDH12-HA (PV input n = 18 cells, CCK inputs n = 20 cells, two-sided Wilcoxon paired test p = 0.2114) and CDH13-HA proteins (PV input n = 41 cells, CCK inputs n = 35 cells, two-sided Wilcoxon paired test ***p< 0.0001) apposed to PV+ and CCK+ boutons. Each dot represents an individual cell. Scale bars: 100µm (**b**), 5µm (**b, c**), 1µm (inserts **c**). Source data are provided as a Source Data file.

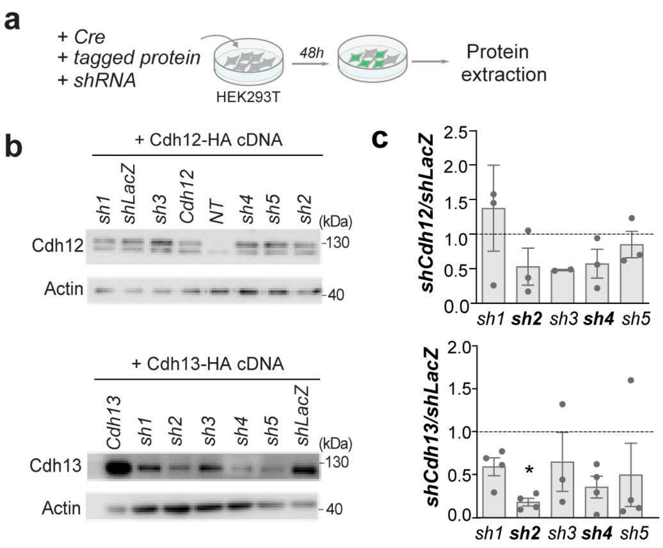

Supplementary Figure 6: In vitro selection of shRNA

a Experimental design of the in vitro shRNA selection process. **b** *In vitro* protein expression of *Cdh12* and *Cdh13* HA-tagged constructs from transfected HEK923T cells assessed by Western blot. Selected shRNAs appear in bold on the graphs. **c** Normalized protein signal for each shRNA (n = 3 independent cultures). Each dot represents an individual replicate. Data are represented as mean ± SEM. Source data are provided as a Source Data file.

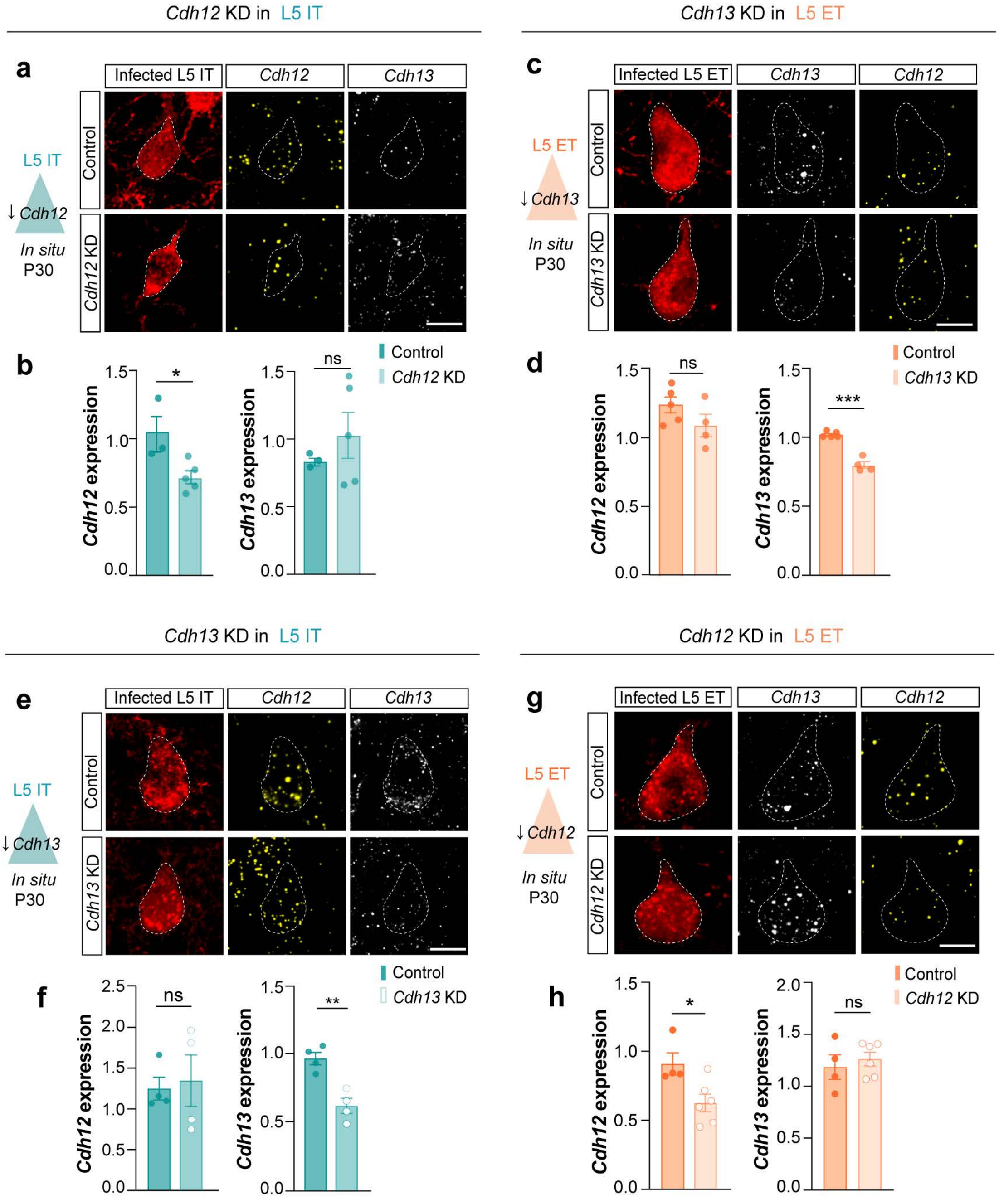

Supplementary Figure 7: *In vivo* validation of *Cdh12* and *Cdh13* down-regulation

a, e Representative images of *Cdh12* (yellow) and *Cdh13* (white) RNA expression at P30 in control, *Cdh12* KD (**a**) or *Cdh13* KD (**e**) L5 IT-expressing cells. **b** Normalized *Cdh12* (Control *n* = 3 mice, 86 cells; *Cdh12* KD *n* = 5 mice, 154 cells; two-sided t-test **p* = 0.0177) and *Cdh13* RNA expression (Control *n* = 3 mice, 82 cells; *Cdh12* KD *n* = 5 mice, 152 cells; two-sided t-test *p* = 0.4122) in control or *Cdh12* KD L5 IT neurons. **d** Normalized *Cdh12* (Control *n* = 4 mice, 120 cells; *Cdh13* KD *n* = 4 mice, 88 cells; t-test *p* = 0.7870) and *Cdh13* RNA expression (Control *n* = 3 mice, 126 cells; *Cdh12* KD *n* = 5 mice, 105 cells; two-sided t-test ***p* = 0.0033) in control or *Cdh13* KD L5 ET neurons. **c, g** Representative images of *Cdh12* (yellow) and *Cdh13* (white) RNA expression at P30 in control, *Cdh12* KD (**g**) or *Cdh13* KD (**c**) L5 ET-expressing neurons. **f** Normalized *Cdh12* (Control *n* = 5 mice, 82 cells; *Cdh13* KD *n* = 4 mice, 48 cells) and *Cdh13* RNA expression (Control n = 5 mice, 88 cells; Cdh13 KD *n* = 4 mice, 50 cells; two-sided t-test ****p*<0.0001) in control or *Cdh13* KD L5 IT-expressing neurons. **h** Normalized *Cdh12* (Control n = 4 mice, 194 cells; *Cdh12* KD *n* = 6 mice, 228 cells; two-sided t-test *p = 0.0306) and *Cdh13* RNA expression (Control *n* = 4 mice, 177 cells; *Cdh12* KD *n* = 6 mice, 219 cells; two-sided t-test p = 0.5960) in control or *Cdh12* KD L5 ET-expressing neurons. Each dot represents an individual animal. Data are represented as mean ± SEM. Scale bars: 10µm (**a, c, e, g**). Source data are provided as a Source Data file.

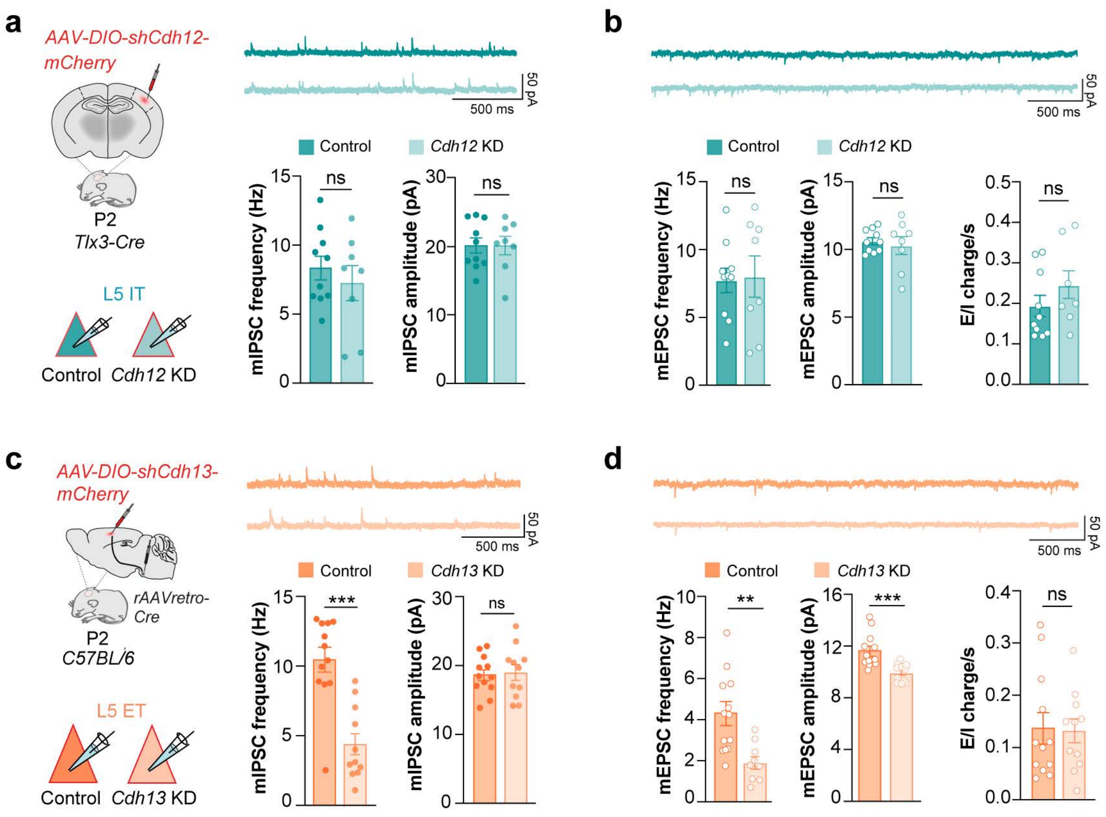

Supplementary Figure 8: Functional synaptic outcome of *Cdh12* KD in L5 IT neurons and *Cdh13* KD in L5 ET neurons

a Experimental design. Example traces of mIPSCs recorded from control or *Cdh12* KD L5 IT-expressing cells. mIPSC frequency and amplitude from L5 IT cells in both conditions (Control n = 10 cells, 6 mice, *Cdh12* KD n = 8 cells, 5 mice; mIPSC frequency two-sided t-test p = 0.4645; mIPSC amplitude two-sided t-test p = 0.9895). **b** Example traces of mEPSCs recorded from control or *Cdh12* KD L5 IT-expressing cells. mEPSC frequency, amplitude and E/I charge from L5 IT cells in both conditions (Control n = 10 cells, 5 mice, *Cdh12* KD n = 8 cells, 5 mice; mEPSC frequency two-sided t-test p = 0.8711; mEPSC amplitude two-sided t-test p = 0.6293; E/I t-test p = 0.2333). **c** Experimental design. Example traces of mIPSCs recorded from control or *Cdh13* KD L5 ET-expressing cells. mIPSC frequency and amplitude from L5 ET cells in both conditions (Control n = 12 cells, 6 mice, *Cdh13* KD n = 11 cells, 6 mice; mIPSC frequency two-sided t-test ***p< 0.0001; mIPSC amplitude two-sided t-test p = 0.7736). **d** Example traces of mEPSCs recorded from control or *Cdh13* KD L5 ET-expressing cells. mEPSC frequency, amplitude and E/I charge from L5 ET cells in both conditions (Control n = 12 cells, 7 mice, *Cdh13* KD n = 10 cells, 7 mice; mEPSC frequency two-sided t-test **p = 0.0029; mEPSC amplitude two-sided t-test ***p = 0.0009; E/I t-test p = 0.8790). Each dot represents an individual cell. Data are mean ± SEM, ns, not significant. Source data are provided as a Source Data file.

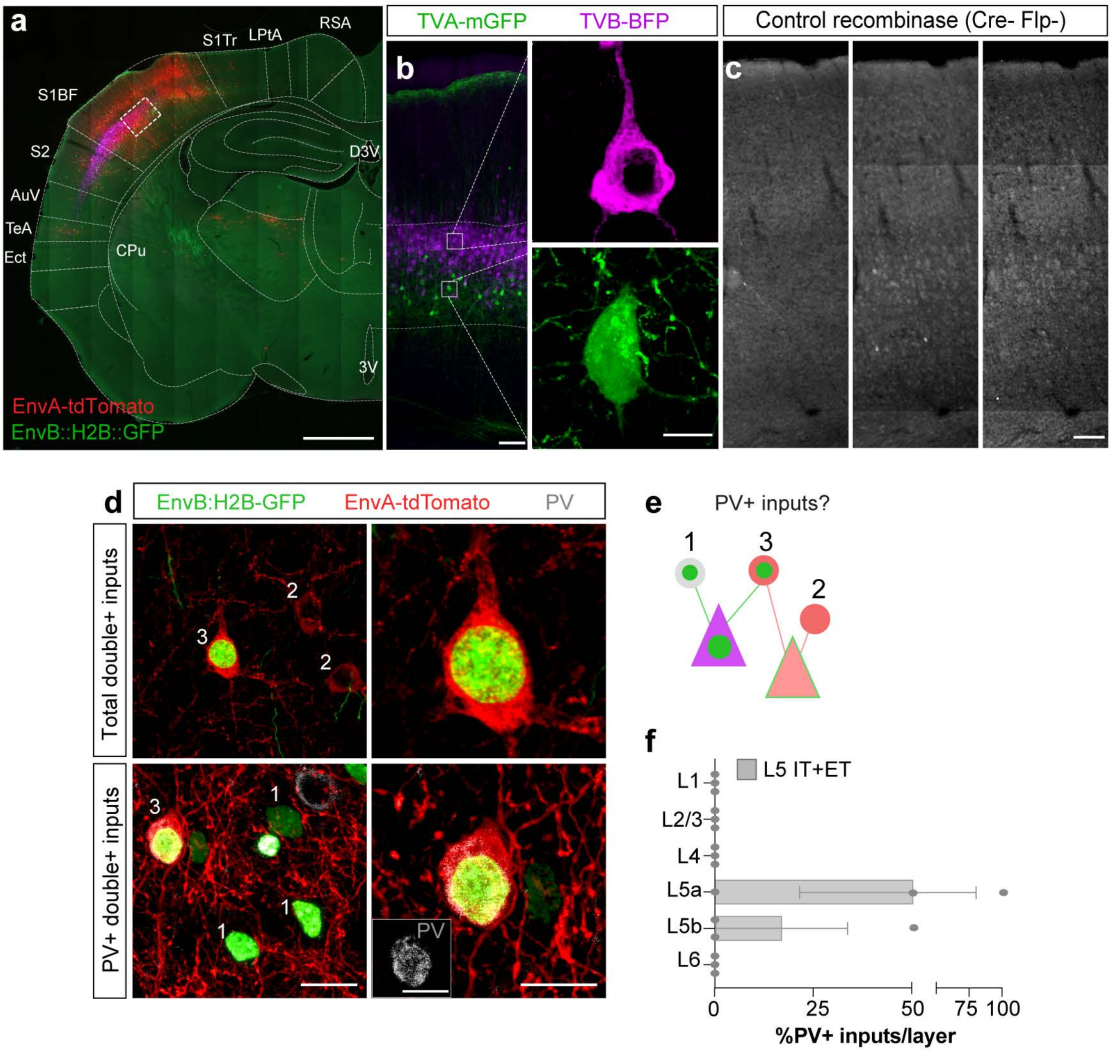

Supplementary Figure 9: Cell type-specific expression of the multiplex monosynaptic tracing strategy

a Coronal section depicting TVA/TVB-expressing AAVs and EnvA/EnvB-expressing RV injection sites. **b** Representative images of L5 IT and L5 ET receptor-expressing cells distribution. L5 IT neurons expressing TVB-BFP (magenta) were mainly present in L5a, while L5 ET expressing TVA-mGFP (green) were exclusively found in L5b. **c** Representative images illustrating the absence of receptor- or RV-infected cells in a Cre- and Flp-negative brain. **d** Confocal images illustrating L5 IT (type 2, EnvB-H2B:GFP) or L5 ET (type 1, EnvA-tdTomato) PV+ input cells. A minimal fraction of input cells infected both with EnvA and EnvB rabies (type 3, green and red) were also positive for PV (insert). **e** Schematic of the different PV+ inputs labelling combinations. **f** Proportion of PV+ inputs per layer for L5 IT + L5 ET double inputs (*n* = 3 mice; Two-way ANOVA, ns). Each dot represents an individual mouse. Data are represented as mean ± SEM. 3rd ventricle (3V); Secondary auditory cortex, ventral part (AuV); Caudate putamen (CPu); Dorsal 3rd ventricle (D3V); Ectorhinal cortex (Ect); Lateral parietal association cortex (LPtA); Retrosplenial agranular cortex (RSA); Primary somatosensory cortex, barrel field (S1BF); Secondary somatosensory cortex (S2); Temporal association cortex (TeA). Scale bars: 500µm (**a**), 50µm (**b, c**), 10µm (**b, d**). Source data are provided as a Source Data file.
