# Supplementary material for: Cadherins orchestrate specific patterns of perisomatic inhibition onto distinct pyramidal cell populations
Source: Nat Commun. 2025 May 14;16:4481. doi: 10.1038/s41467-025-59635-z (PMC12078473; doi:10.1038/s41467-025-59635-z)
Supplement: Supplementary file 2 — Reporting Summary [file 41467_2025_59635_MOESM2_ESM.pdf]

nature portfolio

Corresponding author(s): Beatriz Rico

Last updated by author(s): Mar 10, 2025

Reporting Summary

Nature Portfolio wishes to improve the reproducibility of the work that we publish. This form provides structure for consistency and transparency in reporting. For further information on Nature Portfolio policies, see our Editorial Policies and the Editorial Policy Checklist.

Statistics

For all statistical analyses, confirm that the following items are present in the figure legend, table legend, main text, or Methods section.

n/a	Confirmed	
☐	☒	The exact sample size (*n*) for each experimental group/condition, given as a discrete number and unit of measurement
☐	☒	A statement on whether measurements were taken from distinct samples or whether the same sample was measured repeatedly
☐	☒	The statistical test(s) used AND whether they are one- or two-sided<br>*Only common tests should be described solely by name; describe more complex techniques in the Methods section.*
☒	☐	A description of all covariates tested
☐	☒	A description of any assumptions or corrections, such as tests of normality and adjustment for multiple comparisons
☐	☒	A full description of the statistical parameters including central tendency (e.g. means) or other basic estimates (e.g. regression coefficient) AND variation (e.g. standard deviation) or associated estimates of uncertainty (e.g. confidence intervals)
☐	☒	For null hypothesis testing, the test statistic (e.g. *F*, *t*, *r*) with confidence intervals, effect sizes, degrees of freedom and *P* value noted<br>*Give P values as exact values whenever suitable.*
☒	☐	For Bayesian analysis, information on the choice of priors and Markov chain Monte Carlo settings
☒	☐	For hierarchical and complex designs, identification of the appropriate level for tests and full reporting of outcomes
☒	☐	Estimates of effect sizes (e.g. Cohen's *d*, Pearson's *r*), indicating how they were calculated

Our web collection on statistics for biologists contains articles on many of the points above.

Software and code

Policy information about availability of computer code

Data collection	Image Studio Lite LI-COR BioSciences https://www.licor.com/bio/products/software/ image_studio_lite/; RRID: SCR_01421<br>LAS AF Leica Microsystems http://www.leica-microsystems.com; RRID: SCR_013673
Data analysis	Image analysis:<br>MATLAB MathWorks https://uk.mathworks.com/products/matlab.html; RRID:SCR_001622<br>Imaris 7.5.2 Bitplane http://www.bitplane.com/; RRID: SCR_007370<br>Fiji ImageJ 1.53t https://fiji.sc/.<br>Huygens Essential version 23.10 (Scientific Volume Imaging, The Netherlands, http://svi.nl)<br><br>Transcriptomics pipeline:<br>R/Bioconductor package DESeq2 R DESeq2 RRID:SCR_015687<br>R/Bioconductor package ClusterProfiler R ClusterProfiler RRID:SCR_016884<br>org.Mm.eg.db org.Mm.eg.db RRID:SCR_023488<br>NextFlow RNASeq pipeline SeqeraLabs https://www.nextflow.io/example4.html<br><br>Electrophysiology: MiniAnalysis SynaptoSoft, Decatur, GA, USA Mini Analysis Program RRID:SCR_002184<br><br>Statistical analysis: GraphPad 9.3.1 GraphPad Software https://www.graphpad.com/ RRID:SCR_000306

All quantification methods using custom scripts are described in Method Details.

For manuscripts utilizing custom algorithms or software that are central to the research but not yet described in published literature, software must be made available to editors and reviewers. We strongly encourage code deposition in a community repository (e.g. GitHub). See the Nature Portfolio guidelines for submitting code & software for further information.

Data

Policy information about availability of data

All manuscripts must include a data availability statement. This statement should provide the following information, where applicable:

- Accession codes, unique identifiers, or web links for publicly available datasets
- A description of any restrictions on data availability
- For clinical datasets or third party data, please ensure that the statement adheres to our policy

All transcriptomic data used will be made publicly available via the Gene Expression Omnibus upon acceptance.

Research involving human participants, their data, or biological material

Policy information about studies with human participants or human data. See also policy information about sex, gender (identity/presentation), and sexual orientation and race, ethnicity and racism.

Reporting on sex and gender	N/A
Reporting on race, ethnicity, or other socially relevant groupings	N/A
Population characteristics	N/A
Recruitment	N/A
Ethics oversight	N/A

Note that full information on the approval of the study protocol must also be provided in the manuscript.

Field-specific reporting

Please select the one below that is the best fit for your research. If you are not sure, read the appropriate sections before making your selection.

[x] Life sciences [ ] Behavioural & social sciences [ ] Ecological, evolutionary & environmental sciences

For a reference copy of the document with all sections, see nature.com/documents/nr-reporting-summary-flat.pdf

Life sciences study design

All studies must disclose on these points even when the disclosure is negative.

Sample size	No statistical method was used to predetermine sample size. Sample sizes were determined empirically according to our previous experiences and the review of similar experiments in litterature (Favuzzi et al., 2019). At least n= 3 mice was used for all samples.
Data exclusions	Outlier cells for perisomatic inputs and in situ hybridization quantifications were identified using the ROUT Method with Q = 1% (GraphPad 9.3.1), and were excluded from the original dataset.
Replication	3 biological replicates per condition were used for RNASeq.<br>Each experiment involved multiple independent litters.<br>In order to ensure reproducibility, we provided detailed methods and reported the number of mice and neurons included in each dataset as well as error bars and p-values for all experiments.
Randomization	Both male and female animals were randomly allocated to experimental groups. Different genetic manipulations (control versus Cdh12 or Cdh13 KD) were randomly determined.
Blinding	Data collection and analysis were not performed blind to the conditions of the experiments. Blinding was made difficult due to the nature of the experiment (specific injections and dissection). We used the same protocols and custom-written codes for collecting and analyzing data.

Reporting for specific materials, systems and methods

We require information from authors about some types of materials, experimental systems and methods used in many studies. Here, indicate whether each material, system or method listed is relevant to your study. If you are not sure if a list item applies to your research, read the appropriate section before selecting a response.

Materials & experimental systems

n/a	Involved in the study
	x Antibodies
	x Eukaryotic cell lines
x	Palaeontology and archaeology
	x Animals and other organisms
x	Clinical data
x	Dual use research of concern
x	Plants

Methods

n/a	Involved in the study
x	ChIP-seq
	x Flow cytometry
x	MRI-based neuroimaging

Antibodies

Antibodies used	Primary antibodies<br>Mouse anti-NeuN - Sigma-Aldrich MAB377<br>Rabbit anti-NeuN - Millipore ABN78<br>Goat anti-CB1R - Frontier Institute CB1-Go-Af450-1<br>Mouse anti-CB1R - SySy 258011<br>Goat anti-mCherry - Antibodies-Online ABIN1440057<br>Rabbit anti-DsRed - Clontech 632496<br>Mouse anti-Syt2 - ZFIN ZDB-ATB-081002-25<br>Guinea pig anti-RFP - SySy 390004<br>Rabbit anti-tagRFP - EvrogenAB233<br>Chicken anti-PV - SySy 195 006<br>Chicken anti-GFP - Aves Lab GFP-1020<br>Rabbit anti-GFP - Molecular Probes A11122<br><br>Secondary antibodies<br>Donkey anti-rabbit 405 - Abcam Ab175652<br>Goat anti-rabbit 405 - Abcam Ab175651<br>Goat anti-mouse IgG1 488 - Molecular Probes A-21121<br>Goat anti-mouse IgG2a 488 - Molecular Probes A-21131<br>Goat anti-mouse IgG2b 488 - Molecular Probes A-21141<br>Goat anti-chicken 488 - Molecular Probes A-11039<br>Donkey anti-goat 488 - Molecular Probes A-11055<br>Donkey anti-rabbit 555 - Molecular Probes A-31572<br>Donkey anti-goat 555 - Molecular Probes A-21432<br>Goat anti-mouse IgG2b 555 - Molecular Probes A-21147<br>Goat anti-mouse IgG2a 647 - Molecular Probes A-21241<br>Goat anti-mouse IgG2b 647 - Molecular Probes A-21242<br>Donkey anti-mouse 647 - Molecular Probes A-31571<br>Goat anti-rat Cy5 - Molecular Probes A-10525<br>Goat anti-chicken 568 - ThermoFisher A-11041<br>Goat anti-chicken biotin - Vector BA-9010<br>Streptavidin 555 - Molecular Probes S32355<br>Chicken anti-HA HRP - Abcam Ab1190<br>Mouse anti-actin HRP - Sigma A3854<br>Further information can be found in the Methods section.
Validation	All antibodies were validated by the companies we obtained them from prior to purchase. Additionally, the antibodies have been validated in previous published work (del Pino et al., 2017; Favuzzi et al., 2019; Exposito-Alonso et al., 2020; Bernard et al., 2022).

Eukaryotic cell lines

Policy information about cell lines and Sex and Gender in Research

Cell line source(s)	HEK293FT - ThermoScientific Cat# R70007<br>B7GG - Dr. T. Karayannis lab (Brain Research Institute, University of Zurich)<br>BHK-EnvA - The Salk Institute of Biological Sciences<br>BHK-EnvB - The Salk Institute of Biological Sciences<br>HEK293-TVA - Dr. T. Karayannis lab (Brain Research Institute, University of Zurich)<br>HEK293-TVB - The Salk Institute of Biological Sciences
Authentication	None of the cell lines used were authenticated
Mycoplasma contamination	The cell lines were not tested for mycoplasma contamination

Commonly misidentified lines
(See ICLAC register)

Name any commonly misidentified cell lines used in the study and provide a rationale for their use.

Animals and other research organisms

Policy information about studies involving animals; ARRIVE guidelines recommended for reporting animal research, and Sex and Gender in Research

Laboratory animals	Tlx3-Cre (C57BL/6J background), Rico lab (Gerfen, Paletzki, & Heintz, 2013)<br>Nex-Cre (C57BL/6J background), Rico lab (Goebbels, Bormuth, Bode, Hermanson, & Schwab, 2006)<br>C57BL/6J, Charles River 000664; RRID: IMSR_JAX:000664<br>CD1, Charles River<br>Tlx3-Cre and Nex-Cre were maintained in a C57BL/6 background (Charles River Laboratories). Animals were housed in groups of up to five littermates and maintained under standard, temperature controlled, laboratory conditions. Mice were kept on a 12:12 light/dark cycle and received water and food ad libitum.
Wild animals	The study did not involve wild animals.
Reporting on sex	Both male and female animals were used, results are not displayed according to sex.
Field-collected samples	The study did not involve samples collected from the field.
Ethics oversight	All animal procedures were approved by the ethical committee (King's College London) and conducted in accordance with European regulations, and Home Office personal and project licenses under the UK Animals (Scientific Procedures) 1986 Act.

Note that full information on the approval of the study protocol must also be provided in the manuscript.

Plants

Seed stocks	*Report on the source of all seed stocks or other plant material used. If applicable, state the seed stock centre and catalogue number. If plant specimens were collected from the field, describe the collection location, date and sampling procedures.*
Novel plant genotypes	*Describe the methods by which all novel plant genotypes were produced. This includes those generated by transgenic approaches, gene editing, chemical/radiation-based mutagenesis and hybridization. For transgenic lines, describe the transformation method, the number of independent lines analyzed and the generation upon which experiments were performed. For gene-edited lines, describe the editor used, the endogenous sequence targeted for editing, the targeting guide RNA sequence (if applicable) and how the editor was applied.*
Authentication	*Describe any authentication procedures for each seed stock used or novel genotype generated. Describe any experiments used to assess the effect of a mutation and, where applicable, how potential secondary effects (e.g. second site T-DNA insertions, mosiacism, off-target gene editing) were examined.*

Flow Cytometry

Plots

Confirm that:

- [x] The axis labels state the marker and fluorochrome used (e.g. CD4-FITC).
- [x] The axis scales are clearly visible. Include numbers along axes only for bottom left plot of group (a 'group' is an analysis of identical markers).
- [ ] All plots are contour plots with outliers or pseudocolor plots.
- [ ] A numerical value for number of cells or percentage (with statistics) is provided.

Methodology

Sample preparation	Non-fixed, samples prepared on the day of the experiment from mouse somatosensory cortex
Instrument	FACS Aria II and III, Becton Dickinson - 85 nozzle
Software	FACSDiva software for acquisition
Cell population abundance	Abundance of the relevant population: 3.4% IT, 1.2% ET. The purity of the samples were determined by sorting again the fluorescence negative neuronal population. The percentage of purity was 99.5%.

Gating strategy

We designed our gating strategy according to the following:
- FCS and SSC parameters were adjusted in order to sort L5 pyramidal neurons with a cell body > 20um
- Doublets were excluded, only singlets were kept
- The live fraction of L5 IT and L5 ET neurons retrogradely labeled with red fluorescent retrobeads (RB555) was finally gated based on DAPI and PE-A intensity such that DAPI negative and RB555 positive cells were sorted.

[x] Tick this box to confirm that a figure exemplifying the gating strategy is provided in the Supplementary Information.
